# Research trends on the relationship between gut microbiota and colorectal cancer: A bibliometric analysis

**DOI:** 10.3389/fcimb.2022.1027448

**Published:** 2023-01-09

**Authors:** Weigen Wu, Yaobin Ouyang, Pan Zheng, Xinbo Xu, Cong He, Chuan Xie, Junbo Hong, Nonghua Lu, Yin Zhu, Nianshuang Li

**Affiliations:** ^1^ Digestive Disease Hospital, The First Affiliated Hospital of Nanchang University, Nanchang, China; ^2^ Department of Gastroenterology, The First Affiliated Hospital of Nanchang University, Nanchang, China; ^3^ First Clinical Medical College, Nanchang University, Nanchang, China

**Keywords:** colorectal cancer, gut microbiota, bibliometric analysis, research trends, hot spots

## Abstract

**Background:**

Colorectal cancer (CRC)is the third most common cancer in the world and the second leading cause of cancer-related deaths, and over the past two decades, many of these researchers have provided a substantial amount of important information on the role of gut microbes in the development and progression of CRC. A causal relationship between the presence of specific microorganisms and CRC development has also been validated. Although a large number of papers related to this area have been published, no bibliometric study has been conducted to review the current state of research in this area and to highlight the research trends and hotspots in this area. This study aims to analyze the current status and future research trends of gut microbiota and CRC through bibliometric analysis.

**Methods:**

Publications from 2001 to 2022 were retrieved from the Web of Science Core Collection database and screened according to inclusion criteria. VOSviewer and CiteSpace software were used to visualize the research trends in this field, including the analysis of title, country, institution, author, number of publications, year of publication, number of citations, journal, and H-index.

**Results:**

A total of 863 studies were eventually identified, and the articles retrieved were cited an average of 44.85 times each. The number of publications on this topic has been increased steadily since 2011. China and the USA have made the largest contribution in the field. *FRONTIERS IN MICROBIOLOGY* is the top productive journal with 26 papers, and *Gut* journal has the highest average citation (167.23). Shanghai Jiao Tong University is the most contributive institution. Professor Yu J, Sung, Joseph J. Y and Fang JY are the most productive authors in this field. Keyword co-occurrence analysis showed that the terms of “Gut Microbiota”, “Colorectal Cancer”, “Inflammation”, “Probiotic” and “Fusobacterium Nucleatum” were the most frequent, which revealed the research hotpots and trends in this field.

**Conclusions:**

There has been a growing number of publications over the past two decades according to the global trends. China and the USA still maintained the leading position in this field. However, collaboration between institutions needs to be strengthened. It’s commended to pay attention to the latest hotspots, such as “*F. nucleatum*” and “probiotics”. This bibliometric analysis evaluates the scope and trends of gut microbiota and CRC, providing a useful perspective on current research and future directions for studying the link between the gut microbiota and CRC.

## Introduction

Colorectal cancer (CRC) is the third most common cancer and the second leading cause of cancer related deaths in the world, with an estimated number of 1.8 million new cases and about 881,000 deaths worldwide in 2018 (Baidoun et al., 2021). With its continued progression in western countries, the incidence of CRC is predicted to increase to 2.2 million new cases and 1.1 million deaths worldwide by 2030 (Arnold et al., 2017). In China, over 376,000 new cases and 191,000 deaths are estimated to occur annually (Chen et al., 2016). Despite regional differences and declining trends, its burden remains high due to population growth. Several environmental factors have been linked to CRC, such as overweight, western dietary habits, smoking and alcohol use (Bultman, 2017). Additionally, it is well known that genetic mutations and epigenetic alterations are implicated in CRC initiation and progression (De Rosa et al., 2015; Valle et al., 2019). Accumulating evidence has shown that gut microbiota dysbiosis is closely associated with CRC (Song et al., 2020).

The human microbiota comprises trillions of microbes, and the relationship between cancer and microbiota is very complex (Park et al., 2021). Microbiota refers to collective microbial community that live in specific environments, including bacteria, fungi, viruses, and protozoa (Corley and Kubo, 2004). With the evolution of technologies such as cell culture, metagenomics and metabolomics, increasing findings during the past two decades have shown that gut microbiota is crucial for host metabolic health and immune homeostasis. Aberrant gut microbiota could contribute to the development of chronic metabolic diseases such as obesity, inflammatory bowel disease (IBD), and nonalcoholic fatty liver disease (NAFLD) (Fan and Pedersen, 2021). There is growing evidence that dysbiosis of the gut microbiota is significantly linked to the development and progression of CRC (Brennan and Garrett, 2016; Tilg et al., 2018; Yachida et al., 2019). Current research has identified several mechanisms by which gut microbiota dysfunction drives CRC development, for example, aberrant immune response, inflammation, DNA damage, disruption of cell metabolism and proliferation (Wong and Yu, 2019). The patients with CRC harbor distinctive intestinal microbiota compositions, compared to adjacent tissues or healthy controls. In recent years, experimental evidence has identified several specific bacteria species involved in CRC. Generally, the bacteria species including *Fusobacterium Nucleatum* (*Fn*), *Escherichia coli (E. coli)*, and *Enterotoxigenic Bacteroides fragilis (ETBF)* have been identified as the pathogenic bacteria to drive CRC, while other bacteria, such as *Akkermansia muciniphila (A. muciniphila), Lactobacillus rhamnosus (L. rhamnosus)*, and *Bifidobacterium breve (B. breve)* are known as probiotics and have an inhibitory effect on CRC (Schmitt and Greten, 2021). These studies may help us to provide the potential microbiome marker in the diagnosis, treatment and prognosis of CRC.

Bibliometric analysis is a statistical method based on public literature databases (e.g., Web of Science) to analyze and visualize research trends (Tran et al., 2018). Bibliometric analysis has become one of the most widely used methods to assess the credibility, quality and impact of scholarly work (Luukkonen, 1990; Ellegaard and Wallin, 2015). A large number of publications on microbiota and CRC have been published in recent years; however, no systematic studies have been conducted through bibliometric econometric analysis of the association between the gut microbiota and CRC has not been systematically studied. This bibliometric analysis could help researchers to understand the current research situation, research trends and research hotspots in this field. In this study, we aimed to uncover emerging trends in articles, journals and keywords performance, collaboration patterns between authors and institutions, and to explore the research hot topics and future directions in the field of microbiota and CRC through a bibliometric analysis.

## Methods

### Search strategy

In this study, we used Web of Science database (Core Collection) as the data source. All potentially relevant publications were collected based on title (TI) and abstract (AB), and the search strategy was as follows:#1: [TI=(colorectal* OR colon* OR rectum* OR rectal*) OR AB=(colorectal* OR colon* OR rectum* OR rectal*); #2: TI = (cancer* OR neoplasm* OR carcinoma* OR adenoma*) OR AB=(cancer* OR neoplasm* OR carcinoma* OR adenoma*); #3: TI=(microbiota* OR microbiome* OR flora* OR microflora* OR bacteria* OR prebiotic* OR probiotic* OR antibiotic* OR dysbiosis* or Saccharomyces* OR Lactobacillus* OR Bifidobacterium* OR Escherichia coli*) OR AB =(microbiota* OR microbiome* OR flora* OR microflora* OR bacteria* OR prebiotic* OR probiotic* OR antibiotic* OR dysbiosis* or Saccharomyces* OR Lactobacillus* OR Bifidobacterium* OR Escherichia coli*); Final dataset: #1 AND #2 AND #3]. In order to capture as many data sources as possible, we use wildcards (*) that can replace any other character and allow keywords with variable endings (Cheng et al., 2022; Cheng et al., 2022). For example, “cancer*” would also return the terms of “cancer” and “cancers.” The search Articles published in English from 2001 (1 January 2001) to 2022 (25 November 2022).

### Study selection

**Figure 1:**
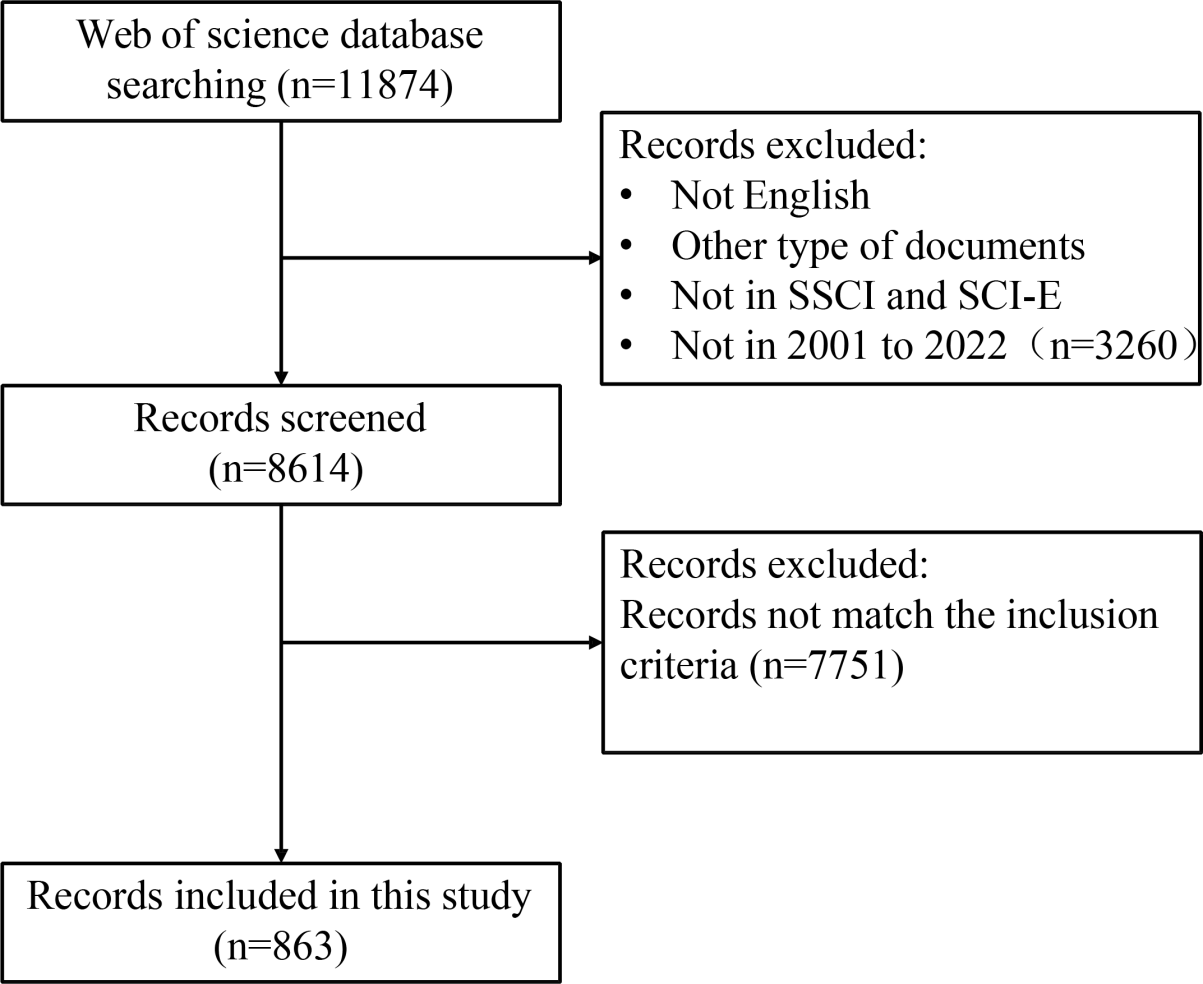
The flowchart of the literature screening process.

The selection criteria and literature screening process of this study was showed in **Figure 1**. Briefly, we entered search terms for an initial search, and then two researchers reviewed the publications identified in the initial search and excluded those that did not fit according to the following inclusion criteria: (1) publication language was limited to “English”; (2) The types of literature included types are articles, but not letter, comments, reviews, or conference abstract; (3) the publication was from the WoSCC Citation Index Expanded (SCI-E) and Social Sciences Citation Index (SSCI) databases; (4) the search time span was from 2001 (1 January 2001) to 2022 (25 November 2022), comprising a total of 21 years. (5) For the selected publication, the subjects of the publication were CRC patients (including CRC patients, preoperative and postoperative CRC patients), CRC animal models, and CRC cellular models, and the studies must also assess the correlation between subjects and gut microbiota. (6) To avoid bias owing to daily updates of the database, we conducted and completed the search and screening of the publication on the same day.

### Data acquisition

Two researchers independently reviewed all included publications and downloaded them, and exported them in different file formats for analysis, extracting the following indicators: the number of publications, frequency of citations, country, institution, journal, author, keywords, Journal impact factor of year 2021 and H-index (defined as the number of papers with citation number > or = H) (Hirsch, 2005).

### Data analysis

VOSviewer (version 1.6.17) was used to create, visualize and explore a collaborative network map of countries, journals, and authors, with each point representing a country/region, institution or author, the number of publications determining the size of the point, and the number of collaborations determining the strength of the links between the points. CiteSpace (version 5.8.R3c) is a visual analysis of temporal trends of keywords.

## Results

### Analysis of publications, citation trends and productive journals

**Figure 2:**
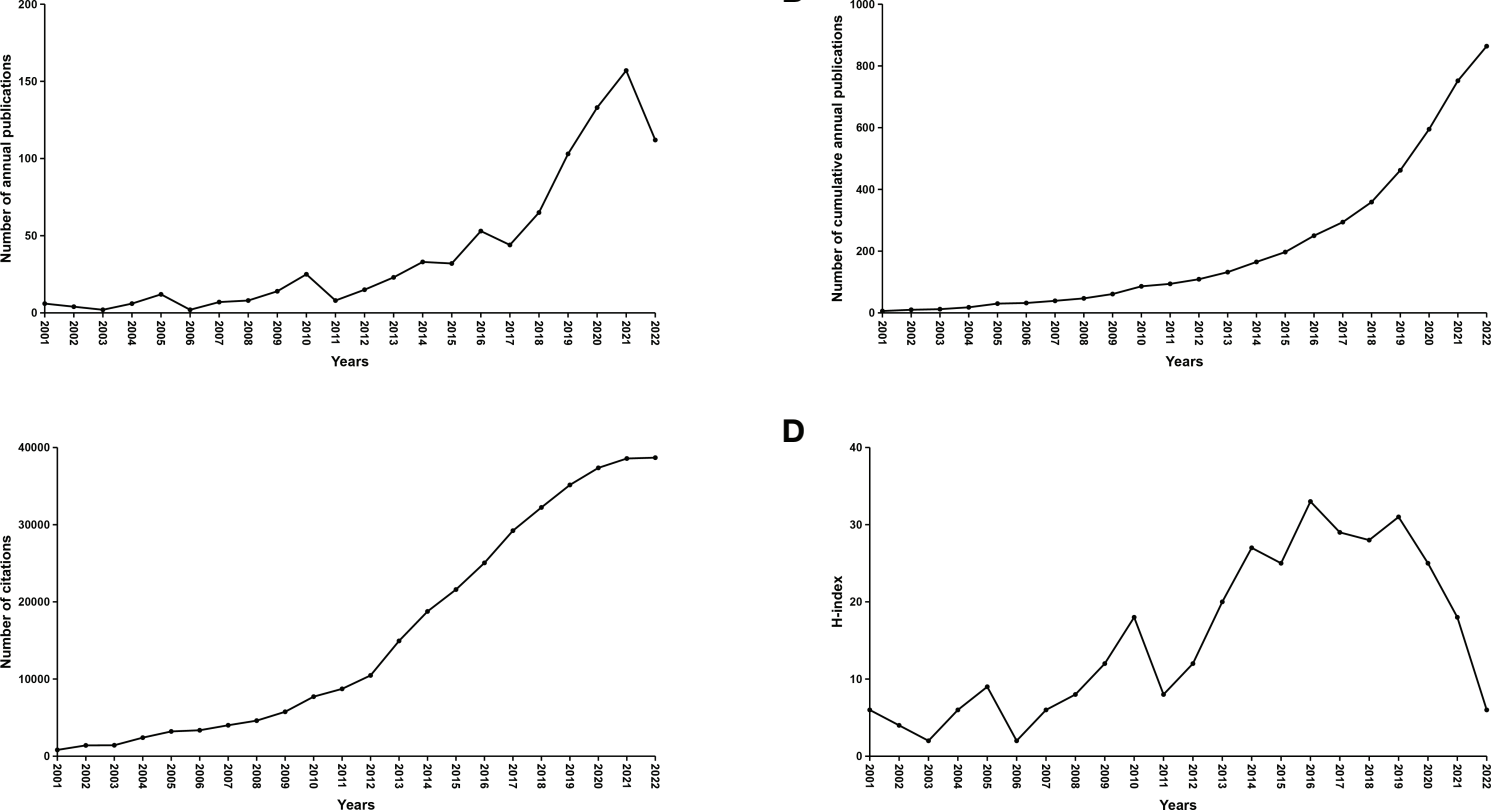
Global number of publications, citations and publication H-index in the field of microbiota and CRC from 2001 to 2022. **(A)** The global annual number of published articles; **(B)** The global number of annual cumulative published articles; **(C)** The global annual number of citations of the publications; **(D)** The global annual H-index values of the publications.

A total of 11,874 records from January 1,2001 to November 25, 2022 were identified. 3,260 records were excluded because the type of literature was not research articles (reviews, conference abstracts, letters, and ongoing papers). The remaining 8,614 records were further assessed by abstract or full-text reading. Finally, 863 studies that met the inclusion and exclusion criteria were included in this bibliometric analysis (**Figure 1**). The number of annual and cumulative publications has increased significantly over the last 21 years. In 2021, the number of publications reached a peak (157) (**Figures 2A, B**). The number of citations has been gradually increasing from 2001 to 2021. Since 2009, the number of citations has overgrown. In 2012, it has reached a high value (more than 10,000 total citations) (**Figure 2C**). For the annual H-index of the field, it was relatively low (<10) until 2008 and increased rapidly after 2009, reaching a peak in 2014-2019 (H-index: 25-31). From 2020 to 2021, the annual H-index decreases slightly, and the significant decrease in 2021 may be related to the delay in indexation (**Figure 2D**). These findings suggest that the relationship between microbiota and CRC has drawn increasing attention in recent years and that future research in this area may become a global hotspot.

Next, the journals studied were analyzed for papers published between 2001 and 2022. In total, 863 papers were published in 340 journals. The top ten most prolific journals are listed in **Table 1**. The most prolific journal was *FRONTIERS IN MICROBIOLOGY* (26), followed by *PLOS ONE* (23) and *SCIENTIFIC REPORTS* (21). Although *Gut* ranks 10th in number of publications, it has the highest IF (31.795) and average number of citations (167.23) of the ten journals, making it the most influential journal in the field.

**Table 1:** The top 12 leading journals in the field of microbiota and colorectal cancer research from 2001-2021.

Journal	Publications	Citations	Citations per-publication	H-index	Journal IF (2021)
FRONTIERS IN MICROBIOLOGY	26	785	30.19	13	6.064
PLOS ONE	23	2481	107.87	17	3.752
SCIENTIFIC REPORTS	21	463	22.05	12	4.997
FRONTIERS IN ONCOLOGY	18	127	7.06	7	5.738
JOURNAL OF FUNCTIONAL FOODS	15	274	18.27	9	5.223
NUTRITION AND CANCER AN INTERNATIONAL JOURNAL	14	581	41.5	11	2.816
WORLD JOURNAL OF GASTROENTEROLOGY	14	898	64.14	10	5.374
CANCERS	13	108	8.31	6	6.575
FRONTIERS IN CELLULAR AND INFECTION MICROBIOLOGY	13	197	15.15	6	6.073
GUT	13	2174	167.23	12	31.795

### Countries/regions analysis

A total of 66 countries/regions have published studies on microbiota and CRC. **Table S1** shows the top 10 most prolific countries. China published the most papers with 334, followed by the USA (166), South Korea (56) and Japan (50). In terms of H-index, the United States (61), China (50) and Japan (24) were the countries with the highest H-index. The United States had the highest citation rate of 14,881, followed by China (11,946) and France (4,138). And France (118.23), the United States (89.65) and Japan (64.10) are the countries with the highest average number of citations.

**Figure 3:**
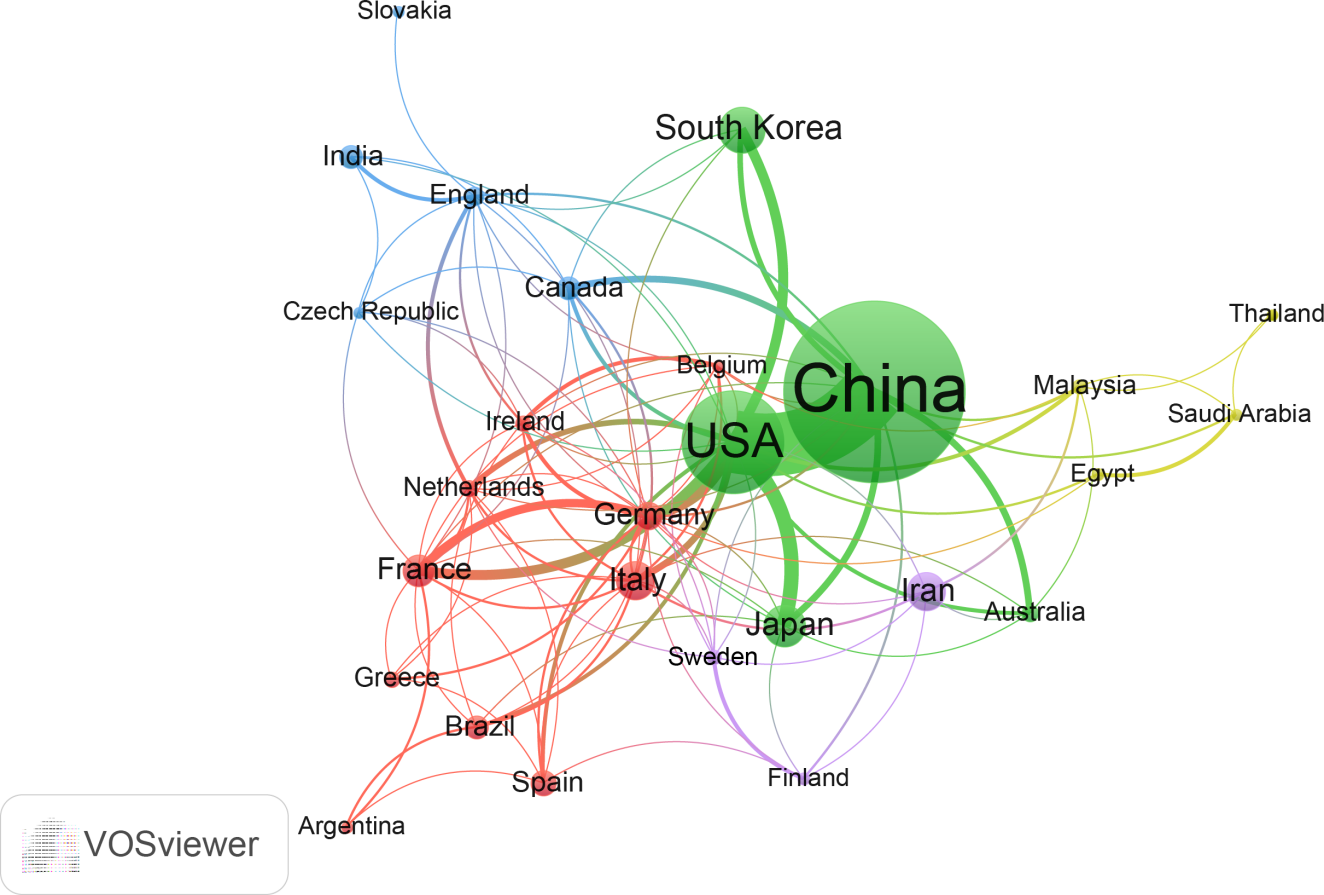
The cooperation network of countries/regions in the field. Dots represent countries, with larger dots indicating a high number of publications, clusters are marked using different colors and links represent cooperation between countries.

The network of cooperation among these countries is shown in **Figure 3**. All countries have no less than seven publications in this area, and a total of 28 countries are within this network map. Among them, China and the United States are arguably the most central countries in the network and the two most closely connected to each other. In addition, the U.S. has some links to almost all countries in the network, with stronger links to Japan, Korea, Germany and France. Compared to the United States, China is less connected to other countries and has some connections with Japan, South Korea, Australia, Canada and other countries.

### Contribution of institutions and authors

**Figure 4:**
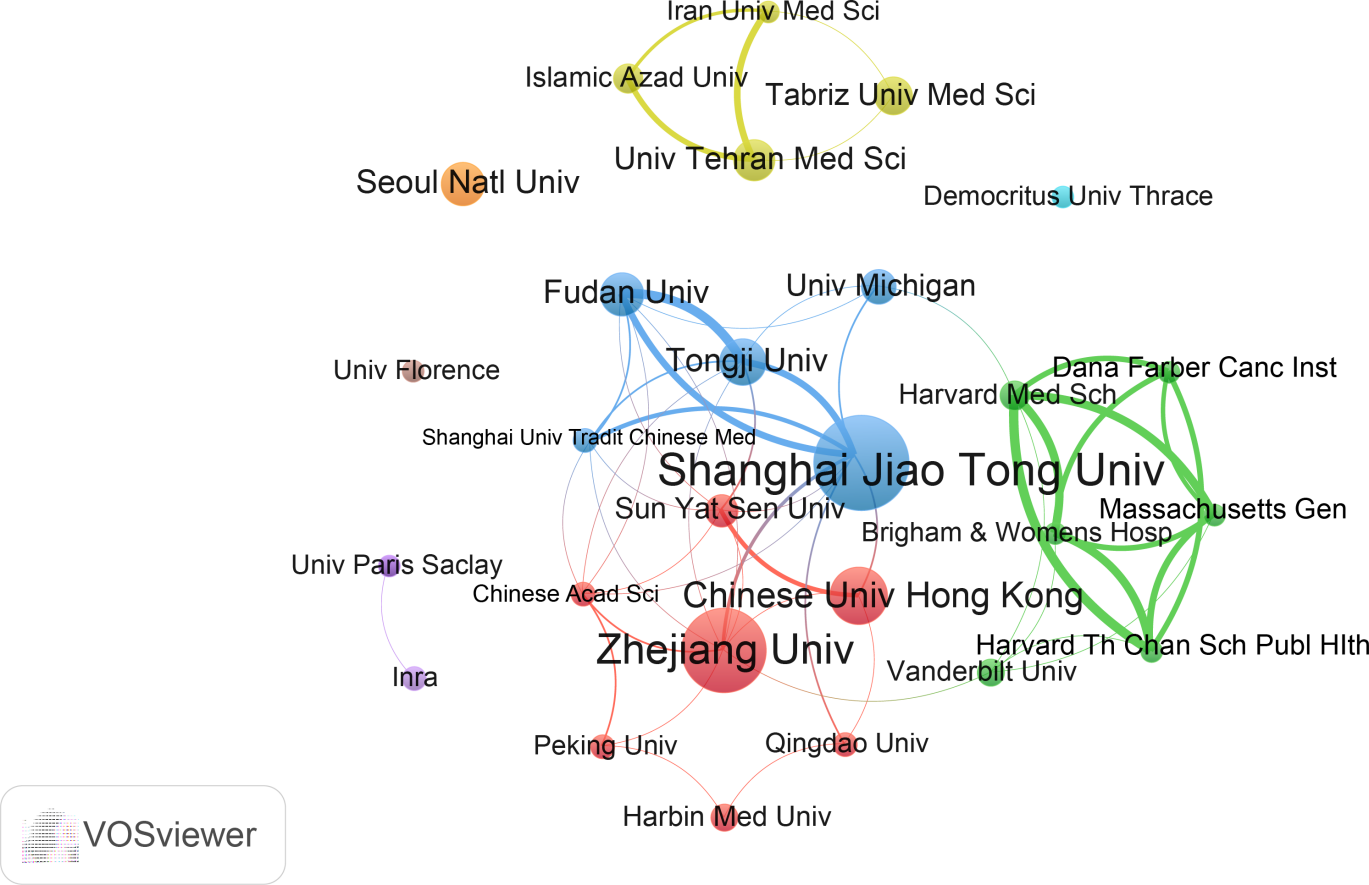
The cooperation network of institutions in the field. Dots represent institutions, with larger dots indicating a high number of publications, clusters are marked using different colors and links represent cooperation between institutions.

We evaluated the most productive universities/institutions in the study. As shown in **Table S2**, Shanghai Jiao Tong University contributed the highest number of papers (36), followed by Zhejiang University, Chinese University of Hong Kong, and other institutions. In addition, Shanghai Jiao Tong University was the institution with the highest total number of citations and H-index. The institution with the highest average number of citations was the Michigan University. The collaborative cluster network of institutions is shown in **Figure 4**. A total of 27 universities/institutions are classified into different groups, which are marked with different colors. Shanghai Jiao Tong University and Zhejiang University are the core nodes. In this network, Shanghai Jiao Tong University has links with nine institutions, with the strongest links with Tongji University and Fudan University. Zhejiang University is connected to nine institutions, with the strongest connection to Shanghai Jiao Tong University.

**Figure 5:**
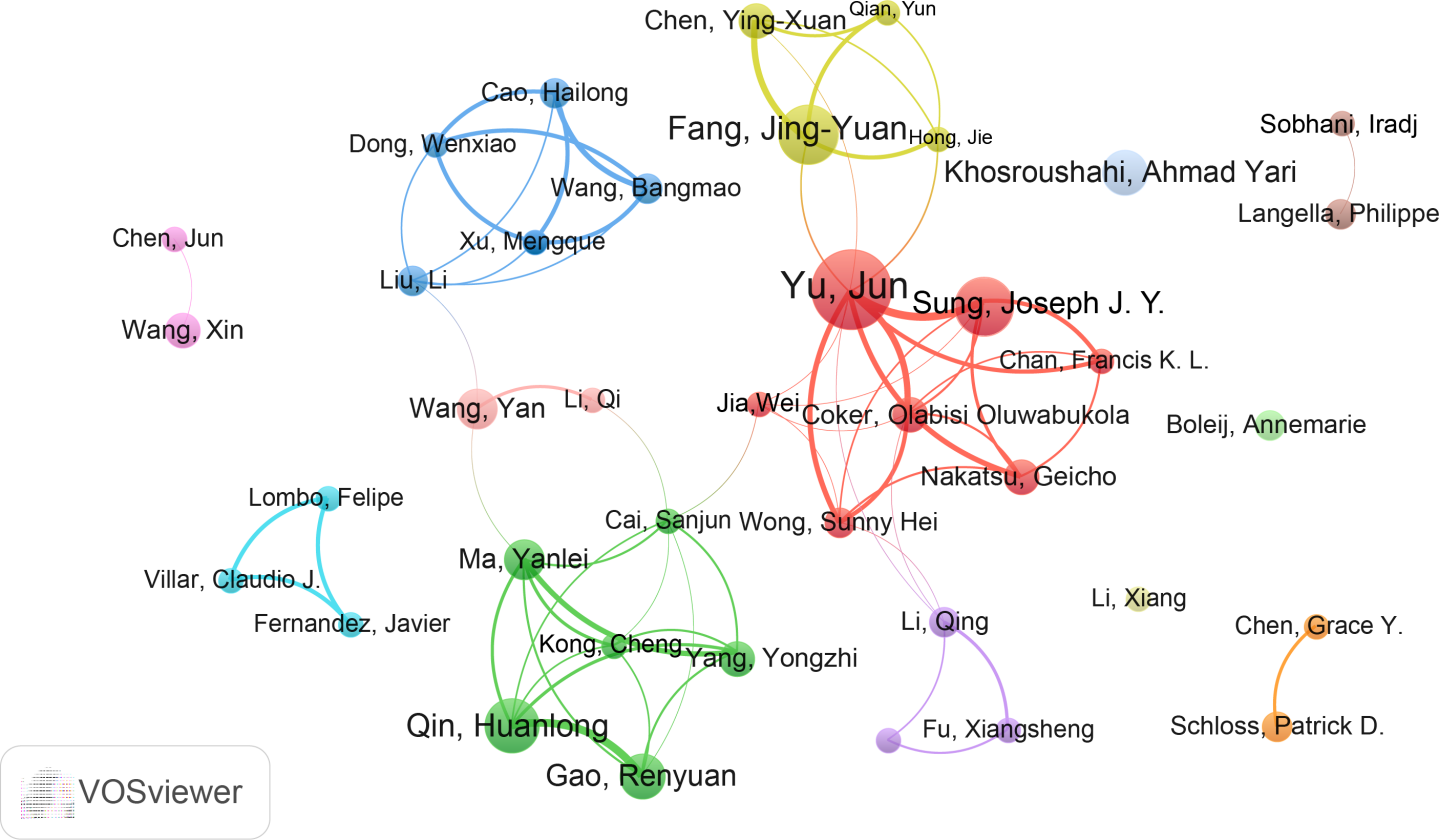
The cooperation network of authors in the field. Dots represent authors, with larger dots indicating a high number of publications, clusters are marked using different colors and links represent cooperation between authors.

Furthermore, we assessed the most productive authors in this study. Professor Yu Jun from the Chinese University of Hong Kong was the most productive author with 17 publications, followed by Sung Joseph J. Y (14) and Fang JingYuan (12). Similarly, Yu Jun had the highest total citations (1655) and H-index (14), while the author with the highest average citations was Sung Joseph J. Y. (106.86) (**Table S3**). The co-authorship analysis was performed by VOSviewer (**Figure 5**). A total of 39 authors were included and they were divided into different groups (colors) based on their collaborations. Six major collaborative clusters were identified in which Yu Jun is the core of the network. Sung Joseph J. Y, Fang JingYuan and Qin Huanlong have a strong influence in this field. While authors such as Li Xiang, Boleij Annemarie, and Khosroushahi Ahmad Yari need more cooperation with these 6 major collaborative clusters.

### Co-cited references and references burst

Of the 863 studies on microbiota and CRC, **Table 2** shows the top 10 research articles ranked by citations from 2001 to 2022 (data from the Web of Science database). The most cited paper was published by Rubinstein and Mara Roxana in Cell. The study found that *F. nucleatum* adheres to, invades, and induces oncogenic and inflammatory responses to stimulate the growth of CRC cells through its unique FadA adhesion (Rubinstein et al., 2013). Furthermore, a paper published by TaChung Yu et al. in 2017 proposed that *F. nucleatum* targeted TLR4 and MYD88 innate immune signaling and specific microRNAs to activate the autophagy pathway and alter CRC chemotherapeutic response (Yu et al., 2017). Eight studies on the impact of gut microbes on CRC development, two of which specifically studied the association of *E. coli* with CRC association and two studies on fecal microbial changes in CRC.

**Table 2:** The top 10 most cited articles in the field of microbiota and colorectal cancer research from 2001-2021.

Rank	Author	Journal	Title	Citations of Web of science	Institutions
1	Rubinstein, Mara Roxana	CELL HOST &MICROBE 2013;14(2):195-206	Fusobacterium nucleatum Promotes Colorectal Carcinogenesis by Modulating E-Cadherin/beta-Catenin Signaling via its FadA Adhesin Fusobacterium nucleatum	1053	Case Western Reserve University
2	Yu, TaChung	CELL 2017;170(3):548-+	Promotes Chemoresistance to Colorectal Cancer by Modulating Autophagy Structural segregation of gut microbiota	802	Shanghai Jiao Tong University
3	Wang, Tingting	ISME JOURNAL 2012,6(2):320-329	between colorectal cancer patients and healthy volunteers	722	Shanghai Jiao Tong University
4	Feng, Qiang	NATURE COMMUNICATIONS 2015:6:6528 MOLECULAR SYSTEMS	Gut microbiome development along the colorectal adenoma-carcinoma sequence	611	BGI-Shenzhen : University Copenhagen
5	Zeller, Georg	BIOLOGY 2014;10(11):766	Potential of fecal microbiota for early-stage detection of colorectal cancer	552	Struct & Computat Biol Unit
6	Martin, HM	GASTROENTEROLOGY 2004;127(1):80-93	Enhanced Escherichia coli adherence and invasion in Crohn's disease and colon cancer	547	University of Liverpool

**Figure 6:**
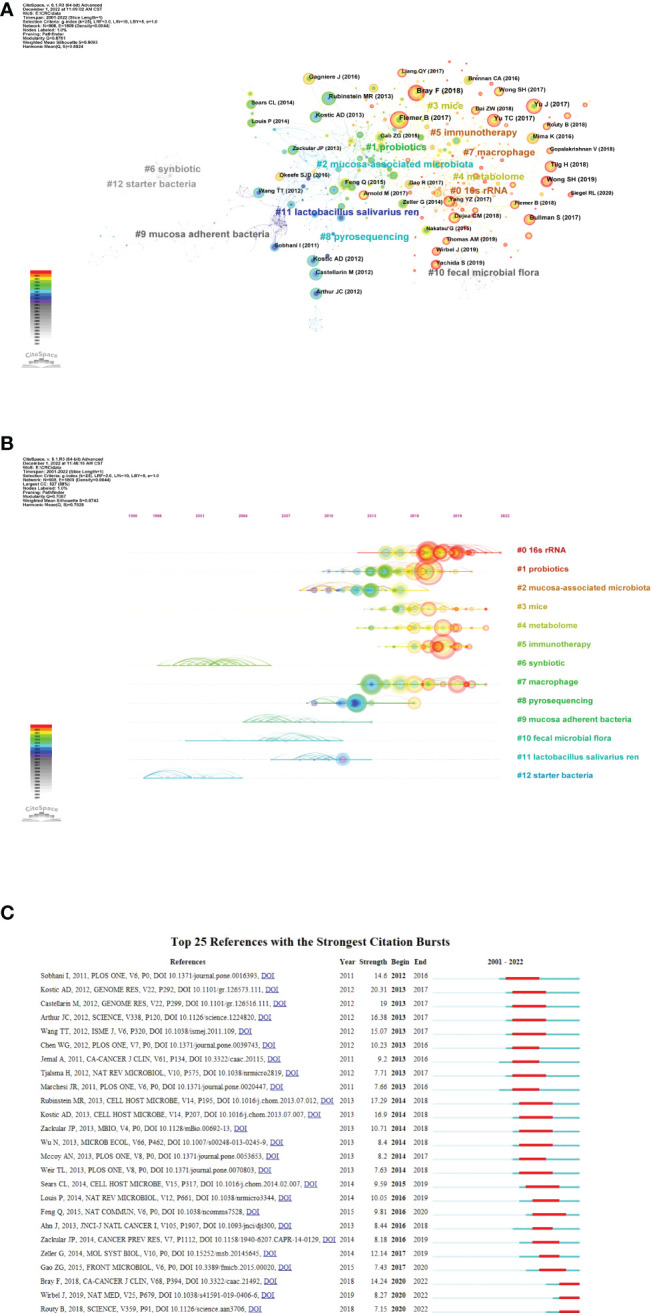
Reference co-citation analysis by CiteSpace. **(A)** Cluster view of reference co-citation **(B)** Timeline view of reference co-citation The clusters are placed vertically in descending order by size. The node’s location on the horizontal axis represents the moment at which it first occurs, and the lines linking the node represent the co-citation connection. The amount of references determines the size of the node. **(C)** Top 25 references with the strongest citation bursts. The blue bar represents the timeline; The red bar represents the burst time period of the references, indicating the start year, end year, and duration of the outbreak.

The references co-citation means that two papers (or more papers) are cited simultaneously by one or more later papers, and then the two papers constitute a co-citation relationship. It is a research method to measure the degree of relationship between references. As shown in **Figure 6A**, the co-citation network can be divided into 12 major subclusters. The modularity Q value is an indicator to evaluate the significance of the cluster structure. A maximum Q value greater than or equal to 0.3 indicates a significant community structure (Wu et al., 2021). In this study, the modularity Q was 0.8761, indicating that the clustering of the network was reasonable. The average profile value was 0.9093, indicating a good homogeneity of clustering. **Figure 6B** showed the timeline view of the co-citation clusters of the reference, which can reflect the temporal characteristics of the research hotspots in the field. The largest cluster is “16s rRNA” (#0), followed by “probiotics” (#1) and “mucosa-associated microbiota” (#2).

Burst references are references that have been widely cited by other studies during a period of time, indicating that they have gotten special attention at a specific time period (Wu et al., 2022). **Figure 6C** shows the top 25 strongest citation bursts between 2001 and 2022. the first to citation burst phenomenon occurred in 2012, which was published by Sobhani et al. in 2011 (Sobhani et al., 2011). The strongest bursts from 2013 came from the 2012 paper by Kostic et al. (2012), followed by Rubinstein and colleagues (Rubinstein et al., 2013). The most recent outburst occurred in 2020 and lasted two years to date.

### Keywords visualization

**Figure 7:**
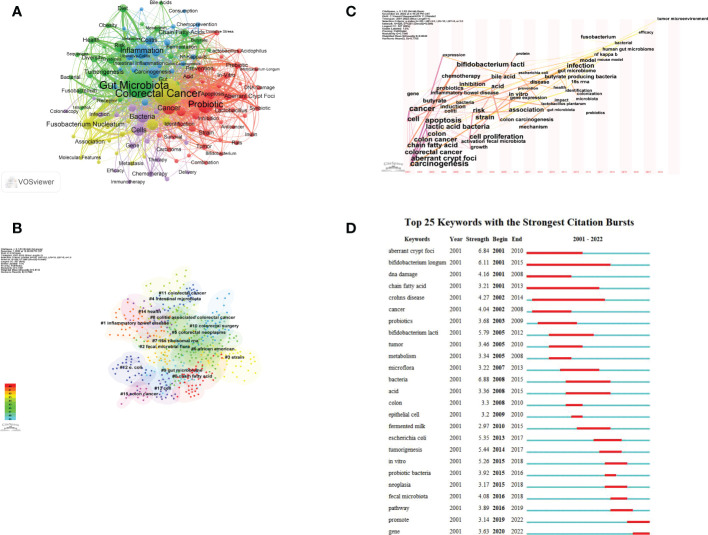
Keywords visualization related to gut microbiota and colorectal cancer research from 2001 to 2022. **(A)** The cooperation network of keywords in the field. Dots represent keywords, with larger dots indicating the frequency of keywords, clusters are marked using different colors and links represent co-occurrence between keywords. **(B)** Keyword clustering analysis in this field. Different colors represent different clusters. Each point represents a keyword and the number on the node represents the cluster the keyword belongs to. The different patterns represented a cluster. Tag# was allocated to clusters, the smaller the count, the more keywords in the cluster. **(C)** Time-based visualization of keyword variation in the field. Dots represented keywords, with larger dots indicated higher occurrence frequency of keywords, the clusters were labeled using different colors, and the links represented the co-occurrence of keywords. **(D)** The top 25 keywords with the strongest citation bursts. A blue bar represents the time period in which the keyword appeared; a red bar represents the interval in which the keyword was found to burst, indicating the start year, the end year and the duration of the burst.

Keyword co-occurrence analysis aims to investigate the co-occurrence relationships among keywords in a group of publications that reflect hot topics. The 863 studies that met the inclusion and exclusion criteria were exported from the WOS database as plain text files, followed by keyword analysis of the 863 articles using VOSviewer with a set threshold of 10. 128 keywords were finally obtained after combining some recurring keywords as well as synonyms. **Figure 7A** shows that “gut microbiota” and “colorectal cancer” were the most prominent keywords. All identified keywords can be divided into 5 clusters: gut microbiota in CRC (yellow). gut microbial metabolites (green), signaling pathways caused by flora and metabolites (blue), probiotics (red), and treatment (purple). These clusters show the most prominent themes in this area of research to date.

**Figure 7B** shows the keyword clustering analysis mapping generated by CiteSpace. A keyword cluster is made up of one or more keywords that have a specific relationship to one another. There have been 15 clustering patterns in total. Most of the clustered keywords and their synonyms also appear in **Figure 7A**, including “ chain fatty acid “ (Cluster0), “inflammatory bowel disease” (Cluster1), “fecal microbial flora” (Cluster2), “strain” (Cluster3), “intestinal microbiota” (Cluster4), “16S rRNA” (Cluster7), “gut microbiome” (Cluster9), “colorectal surgery” (Cluster10), “colorectal cancer” (Cluster11), “e.coli” (Cluster12), “cell” (Cluster13), “health” (Cluster14) and “colon cancer” (Cluster15). And there are many lines between the nodes in these clusters, which means that the domain The co-occurrence of keywords is high.

The keyword time zone map helps us to clearly show the evolution of high frequency keywords, and keyword burst analysis comprises two properties (burst intensity and duration), which can indicate rapid changes in keywords over time and can be utilized as markers of developing research paths. Time zone analysis (**Figure 7C**), and burst analysis (**Figure 7D**) were performed on the keywords by CiteSpace software. Among them, **Figure 7C** set a threshold value of 20, from which 47 keywords were identified. The results of the analysis showed that “CRC”, “abnormal crypt foci”, “chain fatty acids”, “DNA damage”, and “cellular”, “carcinogenic” appeared for the first time in this field. In 2013, “gut microbiota” appeared for the first time. In recent years, specific bacteria such as *Fn* and *E. coli*, 16s rRNA sequencing technology, efficacy, and tumor microenvironment began to appear.

### Specific bacteria linked to CRC

**Figure 8:**
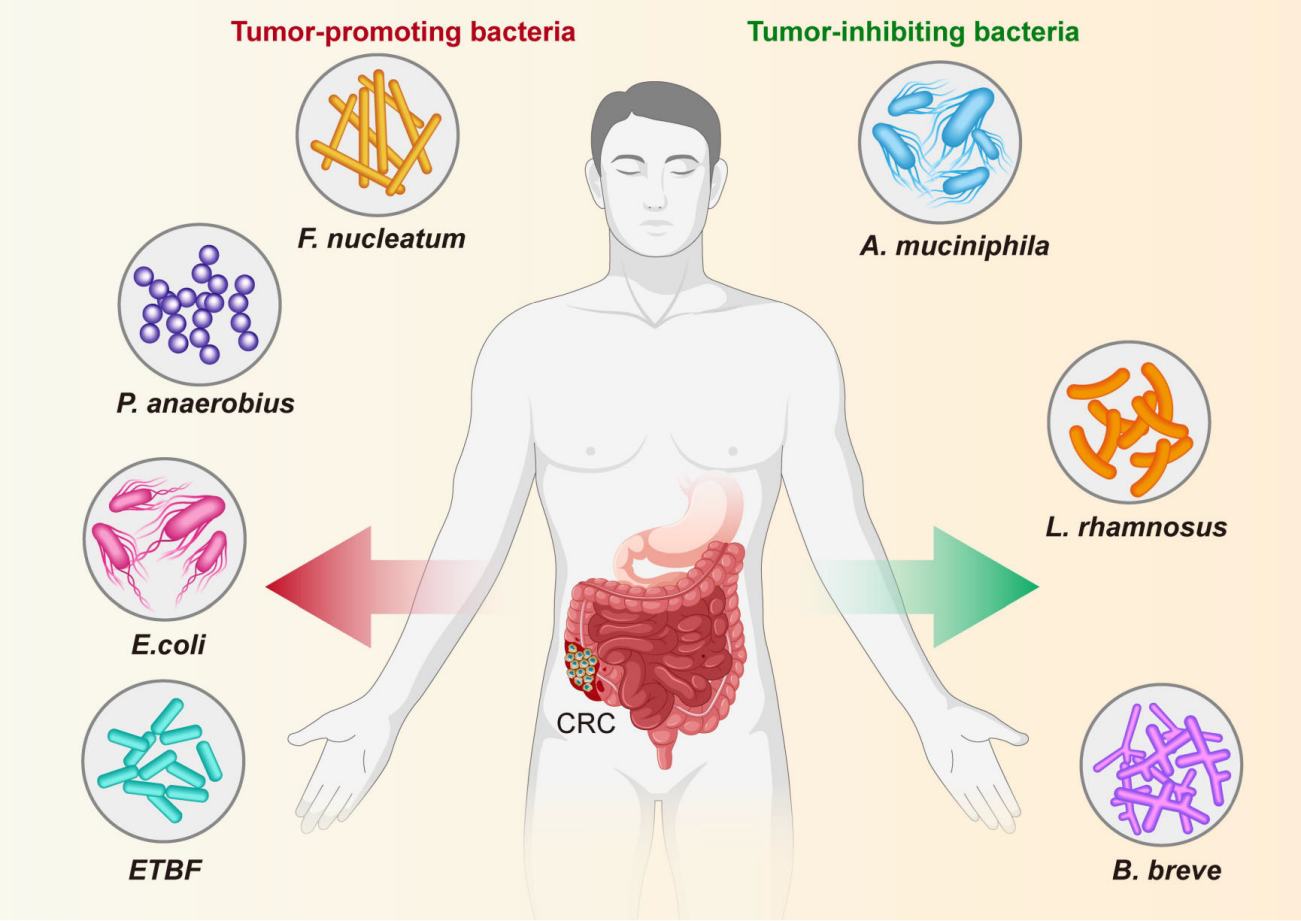
Specific bacteria linked to CRC. The bacteria species including *F nucleatum*, *E coli*, *P. anaerobius* and *ETBF* have been identified as the pathogenic bacteria to drive CRC, while other bacteria, including **(A)** *muciniphila*, *L. rhamnosus*, and **(B)** *breve* are known to have an inhibitory effect on CRC.

For a long time, numerous studies based on sequencing technologies only revealed the alteration of composition and ecology of the gut microbiota in CRC patients or experimental models, the biological function and mechanism by which specific bacteria involved in CRC initiation and progression has not been investigated (**Figure 8**, **Table 3**). Recently, with improvements in culture media, emerging evidence indicates that some specific bacteria pathogens are closely associated with CRC, such as *Fn*, *E. coli*, *ETBF* and *P. anaerobius* (Arthur et al., 2012; Kostic et al., 2013; Tsoi et al., 2017; Chung et al., 2018; Long et al., 2019; Pleguezuelos-Manzano et al., 2020). *Fn* has been a hot topic of research in recent years, with numerous studies conducted at the cellular level, at the animal level and on human specimens (Kostic et al., 2013; Yang et al., 2017; Hashemi Goradel et al., 2019; Rubinstein et al., 2019; Xue et al., 2021; Alturki et al., 2022). In addition, researchers have identified beneficial probiotics such as *Bifidobacterium* and *Lactobacillus* that have anti-inflammatory properties and are capable of producing beneficial metabolites (e.g. short-chain fatty acids) (Molska and Reguła, 2019). In addition, we found that most of the publications screened, studies were conducted using mucosal samples and tended to construct animal models for the studies. Although the number and depth of studies at the mechanistic level have increased in recent years, most studies are still cross-sectional.

**Table 3:** The intestinal bacteria associated with CRC.

Bacterium	Phylum	Function	Study method	Mechanism	Reference
F. nucleatum	Fusobacteria	Cancer promotion	qPCR Metagenomic sequencing	Inflammation response Immune response Cell proliferation Regulation of cell metabolism	(Rubinstein et al., 2013; Yu et al., 2017; Yang et al., 2017; Rubinstein et al., 2019; Kong et al., 2021)
P. anaerobius	Firmicutes	Cancer promotion	Metagenome sequencing Metagenomic sequencing 16S rDNA sequencing	Immune response cell proliferation	(Tsoi et al., 2017; Long et al., 2019)
E. coli	Proteobacteria	Cancer promotion	MPCR	DNA damage	(Cuevas-Ramos et al., 2010; Gagnière et al., 2017)
ETBF	Bacteroidetes	Cancer promotion	qRT-PCR	Inflammation response Immune response Cell proliferation	(Wu et al., 2003; Chung et al., 2018; Hwang et al., 2020)
A. muciniphila	verrucomircrobia	Cancer Inhibition	16S rDNA Gene Sequencing	Immune response	(Howe et al., 2018; Wang et al., 2020)
L. rhamnosus	Firmicutes	Cancer Inhibition	RT-PCR	Immune response	(Owens et al., 2021)
B. breve	Actinobacteria	Cancer Inhibition	qPCR	Immune response Protection of the intestinal barrier	(Yoon et al., 2021)

## Discussion

Studies have shown that the gut microbiota dysfunction is closely associated with CRC, which has resulted in a growing number of studies investigating the relationship between gut microbiome and CRC over the past two decades. However, there has been no bibliometric analysis of the field. In this study, we focused on gut microbiota and CRC findings through manually screening eligible studies and analyzing 863 studies published in the Web of Science database from 2001 to 2022. The increasing number of publications in this field has been driven. With the continuous development of sequencing technology, the researchers could comprehensively dissect the entire gut bacterial community, identify specific bacteria, and gain a better understanding of the overall characteristics of the gut microbiome (Sheflin et al., 2014; Gagnière et al., 2016; Gao et al., 2017). Our quantitative analysis shows that 2011 was a significant year for the field of microbiota and CRC research, as it saw a significant increase in annual publications, total citations and H-index. The temporal trends in total citations and H-index are broadly in line with the number of annual publications, peaking in 2016 (H-index = 33) and declining over the last four years due to the proximity of 2019-2022 to the time of data collection.

We have analyzed the most influential countries, institutions, authors and journals in this area. China and the USA are the two main publishing countries in this field, and these two countries account for 57.9% of all research. China is the most productive country and publishes the most articles, but has a lower H-index, number of citations, and average number of citations than the USA. The disparity in this domain occur may be due to the following reasons: Firstly, given the large land area and population of China, there are many research institutions, resulting in a high volume of publications. Secondly, the incidence of gastrointestinal tumors in China is relatively high, such as gastric and colorectal carcinomas. Notably, the research on the relationship between gut microbiota and colorectal cancers is a hot topic in recent years. In the institution analysis, we discovered that 70.00% of the top 10 productive institutions were situated in China and the USA, demonstrating that publications issued by China and the USA institution were of greater quantity. Research institutions, such as Shanghai Jiao Tong University, are comparatively mature in this research domain and can be considered as an essential institution for cooperation and further learning. According to the journal analysis, the top 10 journals published 19.7%of the articles. The majority of the research was organized into three broad categories: gastrointestinal cancer journals, general interest publications, and microbiological journals. The overall number of articles published in gastrointestinal cancer-related journals was the greatest, indicating that the gut microbiota has become a prominent focus in CRC research.

An analysis of the top 10 most cited articles shows that research has focused on three themes: 1) alterations in the gut microbiota in CRC; 2) alterations in microbial metabolites in CRC; and 3) the association of specific bacteria with CRC. The timeline view of reference co-citation analysis can reflect the dynamic changes and development trends of corresponding clusters in different periods. In **Figure 6B**, Cluster 6 (synbiotic) and cluster 12 (starter bacteria) are the earliest developed, while cluster 0 (16s rRNA) and cluster 5 (immunotherapy) are hot topics. This may mean that the research in this field has passed the macro and superficial stage and gradually expanded to the study of intestinal flora and its metabolites in the prevention, diagnosis and treatment of CRC.

Of the top 25 references with the strongest citation bursts were listed in **Figure 6C**. the first burst came from the publication in 2011 by Sobhani et al., who demonstrated for the first time that microbiota compositional changes in CRC patients may affect mucosal immune responses (Sobhani et al., 2011). The strongest burst from 2013 came from the paper published in 2012 by Kostic et al., followed by Rubinstein et al. Kostic et al., by performing quantitative PCR and 16S rDNA sequence analysis on 95 pairs of CRC/normal DNA, confirmed that *Fusobacterium* sequences were enriched in CRC tissues. However, the role of *Fusobacteria* in the pathogenesis of CRC remains unclear (Kostic et al., 2012). Rubinstein, in turn, shed light on the mechanism by which *Fn* drives CRC (Rubinstein et al., 2013). Interestingly, two papers published in 2018 and one published in 2019 are still in ongoing burst, which means they have received a lot of attention recently.

In terms of the frequency of keywords, we found that “colorectal cancer” and “gut microbiota” were the most prominent keywords. In addition, “Fusobacterium Nucleatum” and “Probiotics” are the latest hotspots at present. From the keyword clustering analysis, keyword time zone diagram and keyword burst analysis, the exploration of CRC and gut microbiota by researchers from 2001 - 2007 was at a relatively macroscopic and superficial stage. Researchers tried to determine the relationship between the gut microbiota and humans, which was mainly a risk factor study. And in the mid-term (2008-2014), the research direction gradually extended to the pathogenesis level and some interactions between CRC and gut microbiota were identified to some extent. In recent years (2015-2022), numerous studies have clarified or identified a number of bacteria that are closely associated with the prevention, diagnosis and treatment of colorectal cancer. However, due to current technical limitations, some bacteria closely related to CRC are difficult to culture and hinder further research. In addition, there are some opinions that certain metabolites produced by the intestinal microbiota are closely related to the diagnosis and treatment of CRC.

The role and potential mechanisms of specific bacteria in the development of colorectal cancer are summarized in **Figure 8** and **Table 3**. *Fn*, a gram-negative anaerobic bacterium, is the most common gut bacterium in CRC (Castellarin et al., 2012). It has been consistently associated at different stages of CRC progression (Kostic et al., 2012; Castellarin et al., 2012; Feng et al., 2015), in different subgroups of CRC (Ito et al., 2015; Yu et al., 2016) and in different ethnic groups (Kostic et al., 2012; Li et al., 2016), and was considered a prognostic biomarker for CRC. *Fn* could be involved in the development of CRC by regulating inflammatory response, immune response, cell proliferation and cell metabolism (Rubinstein et al., 2013; Yu et al., 2017; Yang et al., 2017; Rubinstein et al., 2019; Kong et al., 2021). One study analyzed the *Fn* abundance of 100 CRC tissues and 72 matched normal mucosal tissues by droplet digital PCR and found that the former approached the latter fivefold, with increasing abundance with CRC progression. This result may be predictive of the clinical outcome of CRC patients (Yamaoka et al., 2018). A recent study conducted a macro-genomic association study of the fecal microbiome of 74 CRC patients and 54 controls from China, and further validated biomarkers in an ethnically diverse population. The study found significant enrichment for new species, including *Parvimonas micra* and *Solobacterium moorei*, as well as confirmed associations of *Clostridium perfringens* and oral digestive *streptococci* with CRC. It also highlights the potential of CRC from fecal samples as a non-invasive early diagnostic biomarker (Yu et al., 2017). The underlying mechanism of *P. anaerobius* in CRC development has been also revealed. Tsoi et al. found that *P. anaerobius* increases intracellular ROS levels *via* TLR2 and TLR4, which promotes cholesterol biosynthesis and cell proliferation to CRC (Tsoi et al., 2017). Moreover, *P. anaerobius*, mediated CRC development in *Apc^Min/+^* mice by initiating the PI3K-Akt-nuclear factor-κ light chain enhancer cascade (Long et al., 2019). *ETBF* is found to promote CRC through disruption of the inflammatory response, immune response and cell proliferation (Wu et al., 2003; Chung et al., 2018; Hwang et al., 2020). *E. coli* promoted CRC by producing toxins that have DNA damaging effects (Cuevas-Ramos et al., 2010; Gagnière et al., 2017). Many types of research have demonstrated the role of a specific number of bacteria in the prevention, diagnosis and treatment of CRC. These studies provided new perspectives for further research in this area to follow.

On the other hand, some bacteria, mostly probiotics such as *A. muciniphila*, *L. rhamnosus* and *B. breve*, inhibit the development and progression of CRC by modulating the immune response. A clinical trial found that the use of probiotics may help alleviate gastrointestinal symptoms and post-operative complications in CRC patients (Amitay et al., 2020). Wang et al. reported that *A. muciniphila* was significantly reduced in patients with inflammatory bowel disease, colitis, and colitis-associated CRC mice (Wang et al., 2020). Interestingly, one study showed no significant difference in the amount of *A. muciniphila* between CRC patients and healthy controls (Lopez-Siles et al., 2018). One study even found that *A. muciniphila* promotes the development of CRC (Howe et al., 2018). Joshua et al. showed that *L. rhamnosus* alleviated tumor burden in the murine gut cancer models by a CD8+T cell–dependent manner, suggesting that this strain may be used to enhance the anti-tumor immune response in CRC patients and ultimately increase the breadth and efficacy of immunotherapy (Owens et al., 2021). supplementation with *B. breve* strains has been found to enhance anti-tumor immunity, suggesting it may be strategy to improve the outcome of CRC treatment (Yoon et al., 2021). This suggests that these microorganisms can act as a new therapeutic modality that may have beneficial effects, but is not entirely safe for patients. Larger clinical trials or probiotic mixtures are now needed to confirm efficacy, dosage and interaction with chemotherapeutic agents.

Although we have identified a number of bacteria that are strongly associated with the development of CRC, the culturing of gut microbes in the laboratory is complicated by the fact that most gut microbes are anaerobic, and many strains are killed after only a short exposure to air. This uncultivable nature also hinders the verification and deciphering of the functional properties of the microbiota (Stewart, 2012; Walker et al., 2014). Most genomic studies of gut microbes are currently carried out, but such studies only result in a list of bacteria, and it may miss species that are in low abundance (Lagier et al., 2012). Browne et al. provided a new method (Browne et al., 2016). But it doesn’t address the root of the problem. Therefore, this may be a hot spot for future research. Because bacterial culture is not only desirable but also imperative, this could allow researchers to directly evaluate the interactions between bacteria and host cells, while also studying the connections between bacterial genetics and physiology.

Studies have shown that gut microbial metabolites are associated with CRC. Pejman et al. found that alterations in the gut microbiome might provoke mutations and transform adenomas into carcinomas. These alterations include the secretion of mutagenic metabolites such as H_2_S, NO compounds, spermidine and TMA (trimethylamine), as well as the reduction of butyrate (Salahshouri et al., 2021). Butyrate, as one of the most important members of the short chain fatty acids (SCFAs) family, has anti-inflammatory and antitumor properties through cell metabolism, microbiota homeostasis, immune regulation, and gene epigenetic modulation (Makki et al., 2018). Park et al. reported that butyrate inhibited organ proliferation in CRC patients and enhanced cell death after radiotherapy at the lesion site (Park et al., 2020). This is a potential strategy to minimize the toxicity of radiotherapy and may improve the prognosis of CRC patients. A study suggests that fecal metabolites may be useful for the non-invasive diagnosis of CRC (Coker et al., 2022). In contrast, other gut microbiota metabolites, such as secondary bile acids (SBA), deoxycholic acid (DCA)and lithocholic acid (LCA), promote the development of CRC (Louis et al., 2014). In addition, research has shown that some indicators including SBA/PBA, DCA/CA and LCA/DCA have diagnostic significance for CRC (Nair, 1984; Owen et al., 1987; Imray et al., 1992).

This study also has some limitations. Firstly, the publications we searched were only from the Web of Science database for SCI-E and SSCI, which may have led to the omission of some publications not included in the database. Web of Science is the most widely used database in bibliometric analysis and is built for this type of analysis (Zhang et al., 2020). Meanwhile, The Web of Science database has a strict assessment of publications, which guarantees the high quality of the literature (Shen et al., 2018; Shen et al., 2019; Zhang et al., 2020). Next, we have only introduced English publications in our analysis, which may have missed some non-English studies. Thirdly, the search keyword restriction may have caused some bias. Finally, the search terms may miss some documents.

In conclusion, we assessed and quantified the productivity of global research related to gut microbiota and CRC to present an overall picture of the subject and explore future research directions. The number of publications in this area has snowballed since 2011. China and the USA are the most productive regions. We have also identified this field’s most influential institutions, journals and authors.

## Data availability statement

The original contributions presented in the study are included in the article/**Supplementary Material**. Further inquiries can be directed to the corresponding authors.

## Author contributions

WW, YO and PZ performed the literature search and data extraction. WW, YO and XX performed the statistical analysis. WW and NSL wrote the manuscript. CX, YZ and NSL designed the study. NL and YZ supervised this study. CH, JH and NL modified the manuscript. All authors contributed to the article and approved the submitted version.

## Supplementary material

Supplementary Table 1 The top 10 most productive countries/regions in the field of microbiota and colorectal cancer research from 2001-2021.

Supplementary Table 2 The top 10 most productive institutions in the field of microbiota and colorectal cancer research from 2001-2021.

Supplementary Table 3 The top 10 most productive authors in the field of microbiota and colorectal cancer research from 2001-2021.
