# Supplementary material for: Research trends on the relationship between gut microbiota and colorectal cancer: A bibliometric analysis
Source: Front Cell Infect Microbiol. 2023 Jan 9;12:1027448. doi: 10.3389/fcimb.2022.1027448 (PMC9868464; doi:10.3389/fcimb.2022.1027448)
Supplement: Supplementary Table 1 — The top 10 most productive countries/regions in the field of microbiota and colorectal cancer research from 2001-2021. [file DataSheet_1.pdf]

Table S1: The top 10 most productive countries/regions in the field of microbiota and colorectal cancer research from 2001-2022.

Countries/Regions	Publications	H-index	Citations	Citations per-publication
China	361	50	12538	34.73
USA	166	61	14881	89.65
South Korea	56	23	1779	31.77
Japan	50	24	3205	64.10
Italy	45	19	2004	44.53
Iran	44	17	816	18.55
France	35	21	4138	118.23
Germany	29	16	2692	92.83
Spain	25	14	837	33.48
Brazil	23	14	638	27.57
