# Supplementary material for: Research trends on the relationship between gut microbiota and colorectal cancer: A bibliometric analysis
Source: Front Cell Infect Microbiol. 2023 Jan 9;12:1027448. doi: 10.3389/fcimb.2022.1027448 (PMC9868464; doi:10.3389/fcimb.2022.1027448)
Supplement: Supplementary Table 2 — The top 10 most productive institutions in the field of microbiota and colorectal cancer research from 2001-2021. [file DataSheet_2.pdf]

Table S2: The top 10 most productive institutions in the field of microbiota and colorectal cancer research from 2001-2022.

Institutions	Publications	H-index	Citations	Citations per-publication
Shanghai Jiao Tong University	36	22	3538	98.28
Zhejiang University	31	16	1438	46.39
Chinese University of Hong Kong	21	15	1677	79.86
Tongji University	18	12	965	53.61
Fudan University	16	11	1567	97.94
Seoul National University	16	11	370	23.13
Harvard Medical School	15	11	1268	84.53
Tehran University of Medical Sciences	15	10	221	14.73
Tabriz University of Medical Sciences	14	10	283	20.21
Michigan University	13	12	2615	201.15
