# Supplementary material for: Research trends on the relationship between gut microbiota and colorectal cancer: A bibliometric analysis
Source: Front Cell Infect Microbiol. 2023 Jan 9;12:1027448. doi: 10.3389/fcimb.2022.1027448 (PMC9868464; doi:10.3389/fcimb.2022.1027448)
Supplement: Supplementary Table 3 — The top 10 most productive authors in the field of microbiota and colorectal cancer research from 2001-2021. [file DataSheet_3.pdf]

Table S3: The top 10 most productive scholars in the field of microbiota and colorectal cancer research from 2001-2022.

Author	Publications	H-index	Citations	Citations per-publication
Yu, Jun	17	14	1655	103.44
Sung, Joseph J. Y.	14	12	1496	106.86
Fang, JingYuan	12	8	1272	106.00
Qin, Huanlong	11	10	1094	99.46
Gao, Renyuan	9	8	956	106.22
Khosroushahi, A.Y.	9	7	196	21.78
Ma, Yanlei	8	7	588	73.50
Wang, Yan	8	6	127	15.88
Nakatsu, Geicho	7	5	812	116
Coker, Olabisi Oluwabukola	7	6	375	53.57
